# Iron-based magnetic molecular imprinted polymers and their application in removal and determination of di-*n*-pentyl phthalate in aqueous media

**DOI:** 10.1098/rsos.170672

**Published:** 2017-08-16

**Authors:** Jing Li, Qingxiang Zhou, Yongyong Yuan, Yalin Wu

**Affiliations:** Beijing Key Laboratory of Oil and Gas Pollution Control, College of Geosciences, China University of Petroleum Beijing, Beijing 102249, People's Republic of China

**Keywords:** nanoscale zero-valent iron, molecular imprinted polymers, di-*n*-pentyl phthalate, adsorption, magnetic solid phase extraction

## Abstract

Iron-based magnetic molecular imprinted polymers (Fe@SiO_2_@MIP) were synthesized for highly selective removal and recognition of di-*n*-pentyl phthalate (DnPP) from water. Well-defined core-shell Fe@SiO_2_ nanoparticles (less than 70 nm) were decorated on MIPs reticular layers to endow DnPP-MIPs with magnetic property for the first time. Five other phthalic acid esters including dimethyl phthalate, diethyl phthalate, dipropyl phthalate, di-*n*-butyl phthalate and di-iso-octyl phthalate were used to investigate the adsorptive selectivity to DnPP. The designed experiments were carried out to explore the adsorption kinetics, isotherms and thermodynamics and the results demonstrated that the adsorption was a spontaneous, exothermal and physical adsorption process. The materials were proved to be excellent adsorbents in removal of DnPP with an adsorption capacity as high as 194.15 mg g^−1^ in optimal condition. Furthermore, a magnetic solid phase extraction with Fe@SiO_2_@MIP coupled to high-performance liquid chromatography method was successfully developed for the determination of DnPP, and the proposed method achieved a good linear range of 0.5–250 µg l^−1^ with a correlation coefficient (*R*^2^) of 0.999 and low limit of detection (LOD) of 0.31 µg l^−1^. These materials exhibited excellent capacity in removal and highly sensitive identification of DnPP from aqueous environment samples, and opened a valuable direction for developing new adsorbents for the removal and enrichment of important pollutants.

## 1. Introduction

Phthalic acid esters (PAEs), a kind of endocrine disruption chemicals (EDCs), are always blended in plastic products to enhance the plasticity, or cosmetics to provide versatility [1,2]. Owing to the vast addition of PAEs in plastic or cosmetic products and the wide usage of these products in our daily life, PAEs are ubiquitous in the environment and have been detected in natural water, sewage, sediment, soil and atmospheric particles [3,4]. PAEs can enter into the human body through ingestion, breathing and skin contact [5]. The strong enrichment of PAEs in living organisms throws a potential threat on human health, leading to widespread attention [6,7]. Di-*n*-pentyl phthalate (DnPP) belongs to the family of PAEs. Although it is not been listed in the priority pollutant list, its application in plastic material and resins should not be ignored. Prolonged exposure to DnPP also can lead to adverse effects to living organisms, as reproduction toxicity mainly in males [8,9]. However, little concern is focused on the problem of DnPP nowadays, let alone the study on removal and recognition of DnPP from environmental media.

With respect to the methods to remove PAEs from aqueous media, biological degradation, chemical transformation and physical removal all have been reported in previous research [10,11]. Biodegradation is a green method, but needs a long reaction time [12]. However, microorganisms cannot easily degrade PAEs with large molecular weight from aqueous solution. Chemical degradation processes, like UV–LED/TiO_2_ photocatalytic process, require high energy and cost [13]. Compared with these approaches, more attention has been focused on the adsorption using various adsorbents [14]. Wu's group investigated the adsorption of PAEs by clay [15], and discovered that clay type, compound structure, exchangeable cation and temperature had great influence on the adsorption. Nowadays, molecularly imprinted polymers (MIPs) materials, a new kind of materials, have been reported to remove PAEs from water samples [16]. MIPs show favoured affinity to the template molecule rather than other molecules, and so provide high adsorption ability to target compounds [17]. DnBP attracted the most researchers' attention on MIPs recognition. He's group fabricated DnBP-templated MIPs, and the property was studied for aqueous environment samples analysis [18]. However, the selective removal and detection of DnPP is still an issue to be explored.

Magnetic hybrid materials are prevailing in environmental analysis and remediation fields. Owing to intrinsic magnetic properties, magnetic materials make solid–liquid separation facile under an applied magnetic field to enable the recycling of adsorbents. Therefore the magnetic MIPs composite materials might be potential candidates as novel adsorbents for the removal and detection of PAEs. Nanoscale zero-valent iron particles (NZVIs) are an emerging kind of magnetic materials. Because of the extremely high reactive activity, few researches focused on the physical adsorption of pollutants by NZVIs were found. A lot of work had been conducted to apply NZVIs as reductants to degrade organic pollutants or detoxify heavy metals from water or sludge [19,20]. Perini *et al.* investigated the degradation of ciprofloxacin by NZVIs and found that the reaction was initiated by hydroxyl radical and adsorption was negligible during the process at pH 2.5 [21]. Boparai's group inspected the mechanism of cadmium ion removal by adsorption onto NZVIs and proved it to be chemisorption [22]. However, they did not testify if this process was reversible and if the cadmium ion had a valance change. To our knowledge, the magnetism of NZVIs is comparable with that of the conventional magnetic nanomaterial Fe_3_O_4_ or even stronger than it [23]. So we focused on the application of functionalized NZVIs as stable adsorbents for removal of organic pollutants from environmental matrix. We applied an appropriate protective layer on NZVIs to avoid oxidation and aggregation in aqueous media. Recently, our group demonstrated that polydopamine-functionalized NZVIs can be used as effective adsorbents for removal of polycyclic aromatic hydrocarbons from water [24].

In this paper, we synthesized well-structured ultrafine Fe@SiO_2_ with distinct core-shell layers and unique monodispersity. We modified NZVI particles as a DnPP-MIP layer for the first time to study the adsorption characteristics towards DnPP and the selectivity in comparison with five other PAEs, including DMP, DEP, DPRP, DnBP and DiOP. During this study, several influence factors such as pH value, ionic strength and temperature were investigated. Then adsorption kinetics, adsorption isotherms and thermodynamics were studied for further exploration of the possible mechanism. Besides, their applications as adsorbents in magnetic solid phase extraction (MSPE) towards DnPP from real water samples and drink bottle hot water immersion fluids were also conducted.

## 2. Experimental section

### 2.1. Chemicals and instruments

Standards of dimethyl phthalate (DMP, 99%), diethyl phthalate (DEP, 99%), dipropyl phthalate (DPRP, 99%), di-*n*-butyl phthalate (DnBP,99%), di-*n*-pentyl phthalate (DnPP, 99%), di-iso-octyl phthalate (DiOP, 99%), *N*-isopropylacrylamide (NIPAM, 99%), methacrylic acid (MAA, 98%), azo-iso-butyronitrile (AIBN, 98%), tetraethyl orthosilicate (TEOS, 98%), 3-(trimethoxysilyl)propyl methacrylate (MPS, 97%), ethylene glycol dimethacrylate (EGDMA, 98%), calcium hydride (CaH_2_, AR) and 1-octadecene (95%) were purchased from Aladdin Industrial Co. Ltd (Shanghai, China). HPLC grade methanol and acetonitrile were obtained from J&K Scientific Co. Ltd (Beijing, China). Polyoxyethylene (5) nonylphenylether (Igepal CO-520) was purchased from Aldrich (St Louis, USA). Ferric chloride hexahydrate (FeCl_3_·6H_2_O) was purchased from Sinopharm Chemical Reagent Beijing Co., Ltd. Oleic acid, cyclohexane, ethanol, sodium hydroxide (NaOH), hydrochloric acid (HCl, 37%), ammonia solution (25.0–28.0%) and sodium chloride (NaCl) were purchased from Beijing Chemical Reagent Company (Beijing, China). Acetic acid and acetone were obtained from Guangfu Chemical Reagent Company (Tianjin, China). Ultrapure water was used during our experiments.

Mechanical agitator (Kexi J-J1; Jintan, China), magnetic agitator (Youlian CLT-1A, Jintan, China) and vacuum drying oven (Kewei, Beijing, China) were used to prepare the adsorbent materials. Water-bathing constant temperature vibrator (DSHZ 300A; Taicang, China) was used for adsorption experiment. High-performance liquid chromatography (HPLC) (Agilent 1260; Santa Clara, USA) was used for analysis. The morphology of the nanospheres was investigated by a transmission electron microscope (TEM, JEM-2100 LaB6, Japan), combined with energy-dispersive X-ray spectroscopy (EDS) (Trident XM4, Mahwah, USA) for determination of composition. X-ray diffractometer (XRD) (Bruker AXS D8 FOCUS, Karlsruhe, Germany) was used to analyse the valence state of the iron core. Fourier transform infrared spectroscopy (FT-IR) (8400S, Japan) was used to analyse the surface functional groups. The Brunauer–Emmett–Teller (BET) surface areas were measured by N_2_ adsorption and desorption (Micromeritics ASAP 2020M+C, Norcross, USA). Vibrating sample magnetometer (VSM) (Lake Shore 7410; Westerville, USA) was used for magnetic measurement. UV−vis spectra were collected on a Shimadzu UV-1700PC spectrophotometer (Kyoto, Japan) for the test of thermoresponsive properties.

### 2.2. Synthesis of Fe@SiO_2_@MIP and Fe@SiO_2_@NIP

**Figure 1.**
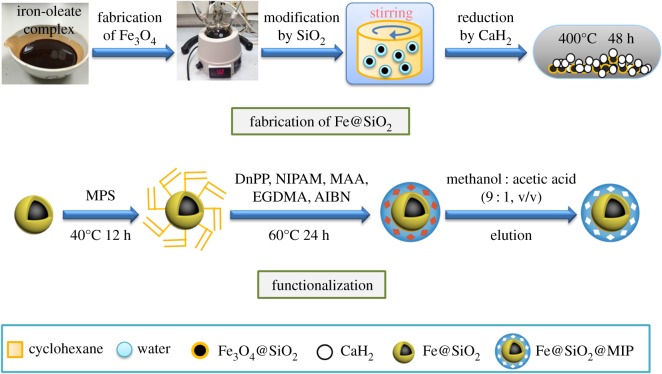
Schematic of the synthesis process.

Fe@SiO_2_ nanoparticles were synthesized through a three-step method. Monodisperse Fe_3_O_4_ nanoparticles were fabricated by the method published by Park *et al.* with minor modifications [25]. Next, a homogeneous coating of SiO_2_ was performed according to water-in-cyclohexane reverse microemulsion method [26]. Then the as-prepared Fe_3_O_4_@SiO_2_ nanoparticles were reduced by CaH_2_ at high temperature (400°C) in a vacuum-sealed glass tube for 48 h [27]. The dispersed Fe@SiO_2_ nanoparticles were then functionalized with MPS [28]. The vinyl groups provided by MPS on the surface of Fe@SiO_2_ could form covalent bonds with MIP polymers. Then MIPs modification was done through seed-precipitation polymerization of comonomers, NIPAM and MAA. The polymer can interact with DnPP through hydrogen-bonding between carbonyl groups and hydrogen atoms in amino and carboxyl groups, which constituted the recognition sites. The prepared materials were designated as Fe@SiO_2_@MIP. The iron-based magnetic non-imprinted polymers (Fe@SiO_2_@NIP) were fabricated under the same conditions without the addition of the template DnPP. The fabrication process is depicted in figure 1 and described in detail in the electronic supplementary material.

### 2.3. Characterization of nanoparticles

TEM analysis was executed at an operating voltage of 200 kV. Their morphologies in different steps were also observed to show the changes in preparation process. Meanwhile, EDS analysis was conducted to analyse the composition. X-ray diffraction (XRD) was performed with Cu Kα radiation (*λ* = 1.5418 Å) at 40 kV and 200 mA in the range of 10–90° (2*θ*) to examine the valence state of Fe. FT-IR was applied to show the functional groups on surface. The BET specific surface areas were measured by nitrogen adsorption at 77 K after out-gassing the samples for 5 h at 80°C. The magnetism (hysteresis loop) was taken on a VSM at a magnetic domain range of −20 000 to 20 000 Oe.

### 2.4. Adsorption experiment

**Figure 2.**
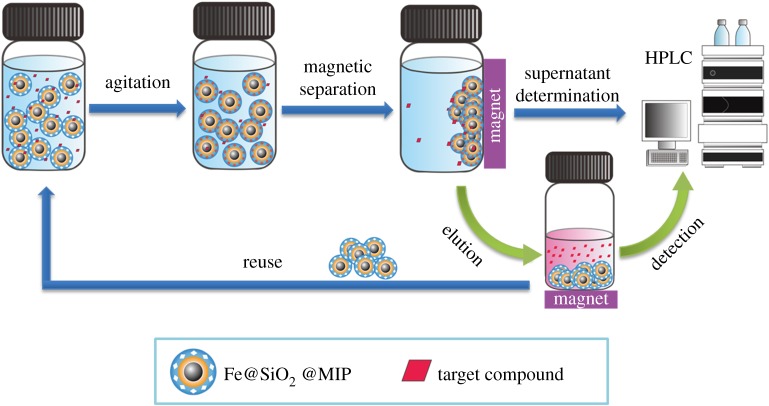
Flowchart of adsorption, enrichment and determination of target compound.

In this work, the Fe@SiO_2_@MIP nanoparticles were used as adsorbents in adsorption experiment for removal of target compound. Meanwhile, the comparison between Fe@SiO_2_@MIP and Fe@SiO_2_@NIP was conducted to study the imprinting effect to template molecular. In order to study the selectivity towards DnPP, the comparison of adsorptions towards different PAEs was also conducted. In this study, parameters of pH value, ionic strength and temperature were also investigated. Batch experiments were conducted in 100 ml polytetrafluoroethylene (PTFE) screw cap vials which were sealed with aluminium foil and contained 10 mg adsorbents. Vials were put into a thermostatic bath at given temperatures with constant agitation (200 r.p.m.) in the dark for a given period. After the set time period, the adsorbents were separated from solution by a magnet. The concentration of the residual targets in the supernatant solution was examined by HPLC. The adsorbents were recycled and reused after a proper elution process. The whole experimental process is shown in figure 2.

The amount of targets adsorbed by the sorbent at time *t* (*Q*_t_, mg g^−1^) and adsorption efficiency (%) were calculated according to the following equations:

2.1 $$Q_{t}=\frac{V\times (C_{0}-C_{t})}{M}$$

and

2.2 $$\text{adsorption efficiency}=\frac{C_{0}-C_{t}}{C_{0}}\times 100\%,$$

where *C*_0_ and *C*_t_ are the concentrations of target compounds in the solution at beginning and time *t* (mg l^−1^), respectively. *V* is the volume of solution (litres); *M* is the mass of sorbent (grams).

Chromatographic analysis was performed using an Agilent 1260 HPLC system. An InertSustain C18 column (4.6 × 250 mm, 5 µm) was used for separation, and the column temperature was maintained at 30°C. The mobile phase was a mixture of acetonitrile and water (90 : 10, v/v) for DnPP, DnBP and DiOP or (80 : 10, v/v) for DMP, DEP and DPRP. The flow rate was controlled at 1 ml min^−1^. Detection wavelength was set at 226 nm.

Adsorption kinetics for extended time periods (5–1200 min) was studied at temperatures of 25°C with a series of independent samples. This was carried out to gauge the adsorption rate and capacity of Fe@SiO_2_@MIP towards DnPP. The experimental kinetic data were fitted to three conventional models, pseudo-first-order model (electronic supplementary material, equation (S1)), pseudo-second-order model (electronic supplementary material, equation (S2)) and intraparticle diffusion model (electronic supplementary material, equation (S3)) [29]. The equation of models are detailed in the electronic supplementary material.

Adsorption isotherms were obtained by varying the initial DnPP concentration from 0.2 to 20 mg l^−1^ in 100 ml vials with 10 mg Fe@SiO_2_@MIP at three temperatures (25, 35 and 45°C). The obtained equilibrium concentration (*q*_e_) at multiple solution temperatures are correlated with several isotherm models (details are available in the electronic supplementary material) [30–33].

The thermodynamic parameters Gibbs free energy (Δ*G*^0^), enthalpy change (Δ*H*^0^) and entropy change (Δ*S*^0^) were used to analyse the sorption mechanism [34]. Several functions were employed to accomplish this analysis [35–37]. Details are shown in the electronic supplementary material.

### 2.5. Analytical methods study

First, 20 mg Fe@SiO_2_@MIP adsorbents were dispersed into 20 ml filtered water samples spiked with target compound at given concentrations. The suspension was vibrated for 3 h at 25°C after 1 min ultrasonic dispersion. After the accomplishment of adsorption, a magnet was put under the bottom of the vials and the supernatant was discarded. Then, the analytes were twice eluted from the nanoparticles with 5 ml methanol. The eluent was separated by a magnet and evaporated to dryness by nitrogen. The residue was re-dissolved with 200 µl methanol and subsequently analysed by HPLC. The whole experimental process is shown in figure 2. The linear range, limit of detection (LOD) and precision were analysed to evaluate the method performance.

Real water samples including Ming Tomb Reservoir and Changping Park water were filtered through a 0.45 µm film and stored at 4°C before use. In addition, hot water extracts of plastic Wahaha bottle and Nongfu Spring bottle were also of consideration. The spiked samples with three different spiked concentration (0, 10, 20 µg l^−1^) were also analysed.

## 3. Results and discussion

### 3.1. Characterization

**Figure 3.**
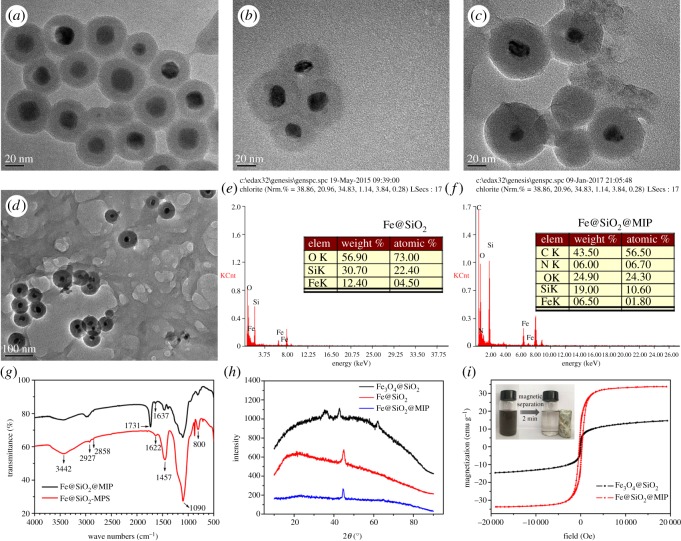
Characterization images. TEM images of (*a*) Fe_3_O_4_@SiO_2_; (*b*) Fe@SiO_2_; (*c,d*) Fe@SiO_2_@MIP; EDS spectrum of (*e*) Fe@SiO_2_ and (*f*) Fe@SiO_2_@MIP; FT-IR spectrum (*g*) of Fe@SiO_2_-MPS and Fe@SiO_2_@MIP; XRD spectrum (*h*) of Fe_3_O_4_@SiO_2_, Fe@SiO_2_ and Fe@SiO_2_@MIP; VSM hysteresis curve (*i*) of Fe@SiO_2_@MIP and the photograph of magnetic separation under magnet.

Figure 3 demonstrates that the as-prepared composites nanomaterials have perfect monodispersed core-shell structure by TEM photographs. Their morphologies in different preparation steps were also observed to show the changes in preparation process. The Fe_3_O_4_@SiO_2_ particles displayed a well-defined core-shell structure, which consisted of a dark core and uniform approximately 16 nm light shell (figure 3*a*). After being reduced by CaH_2_, the cores exhibited a shrink in volume as a result of state change from Fe_3_O_4_ to Fe^0^ (figure 3*b*), which was in accordance with Kohara *et al*.'s report [27]. Obviously, the MIP polymer manifested a network structure, and Fe@SiO_2_ particles were scattered on it (figure 3*c,d*). BET measurement was applied to analyse the potential adsorptive property of adsorbents, which was mainly controlled by particle sizes, porosity and dispersity. Fe@SiO_2_@MIP was found to have a specific surface area of 71.28 m^2^ g^−1^, which was higher than that of Fe@SiO_2_ (58.488 m^2^ g^−1^). It was attributed to the porous mesh structure of MIP polymers.

In figure 3*e,f*, the elements (Fe, Si, O, C and N) and the proportions in Fe@SiO_2_ and Fe@SiO_2_@MIP are listed to make demonstration and comparison. Fe@SiO_2_ mainly comprised Fe, Si and O. The addition of C and N proved MIP polymer existed in Fe@SiO_2_@MIP.

The infrared spectra showed the changes of the surface groups of the particles before and after MIP polymers coating (figure 3*g*). The gradual band centred at 3442 cm^−1^ was attributable to O–H and N–H, and the peaks at 1380 and 3440 cm^−1^ correspond to the hydroxyl groups on the surface of the silica shell [38]. Small peaks at 2927 cm^−1^ and 2858 cm^−1^ showed the existence of the carbon chain, which were more obvious after modification of MIP polymer. Similarly, the emerging C=O peaks at 1731 cm^−1^ and 1637 cm^−1^ in Fe@SiO_2_@MIP sample demonstrated the presence of NIPAM, MAA and EGDMA after polymer modification [39]. The weakening of the C=C peak at 1622, 1457 cm^−1^ and the decrease of Si–O–Si and Si–O peaks at 1090 cm^−1^, 811 cm^−1^ also exhibited the existence of coating on the surface of Fe@SiO_2_-MPS.

Figure 3*h* shows the XRD patterns of the as-prepared composite nanoparticles. The comparison between Fe_3_O_4_@SiO_2_ and Fe@SiO_2_ showed the transformation in valence state after reduction. The presence of magnetite (Fe_3_O_4_) was confirmed by the diffraction peaks at 2*θ* approximately 35°, 43° and 62° [40]. The sharp peak at 44° indicated the existence of Fe^0^. Broad diffraction peaks at 23° depicted the existence of amorphous SiO_2_ [41].

The magnetic properties were determined at room temperature by VSM. The saturation magnetization (Ms) value of Fe@SiO_2_@MIP was found to be 33.75 emu g^−1^, which was enough to separate from water quickly under the additional permanent magnet (figure 3*i*) [42]. This saturation magnetization value was higher than Fe_3_O_4_@MIP (22.6 emu g^−1^) in the study of He's group and mag-MIP beads (5.11 emu g^−1^) in Zhang *et al.*'s research [43,44]. The inset in figure 3*i* provides an intuitive cognition that the adsorbents could be easily separated from water. Besides, the comparison between Fe_3_O_4_@SiO_2_ and Fe@SiO_2_@MIP had confirmed that NZVI had stronger magnetic responsivity than Fe_3_O_4_ which was equal in Fe content [23]. To our delight, the obtained VSM results in conjunction with XRD data indicated that no additional new magnetic phases or oxidized phases had been introduced into the functionalized Fe@SiO_2_, suggesting that the parent Fe@SiO_2_ particles were well retained in the polymer shell during the preparation process.

To be specific, the MIPs displayed no variation of UV–vis response at different temperatures (18–55°C), indicating no thermo-sensitivity (electronic supplementary material, figure S1). It was estimated that the vast amount of cross-linker added in MIPs modification made a firm network, contributing to a stable structure without response to temperature.

### 3.2. Influence of pH, ionic concentration and temperature on adsorption efficiency

**Figure 4.**
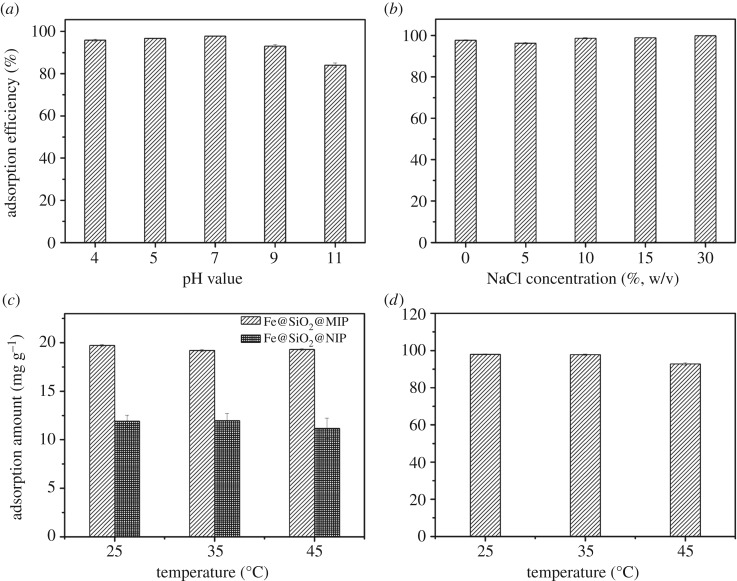
The effect of pH value (*a*), ionic strength (*b*) and temperature (*c,d*) of adsorption solution. ((*c*) Initial concentration 2 mg l^−1^, adsorbent Fe@SiO_2_@MIP and Fe@SiO_2_@NIP and (*d*) initial concentration 10 mg l^−1^, adsorbent Fe@SiO_2_@MIP).

The pH value had influence on adsorption performance (figure 4*a*). It was found that the alkaline condition was obviously not conducive to adsorption. It was because the functional groups in monomers, especially carboxyl in methacrylic acid, were easily affected by alkali condition so as to impede the formation of hydrogen-bonding and inhibited hydrophobic interaction between DnPP and adsorbents. So pH 7 was applied in subsequent experiments.

In this experiment, NaCl was used to adjust the ionic strength, and the addition of NaCl had a slight effect on the adsorption efficiency (figure 4*b*). The difference of adsorption efficiency was not more than 4% in the range of 0–20% (w/v). Along with the increase of NaCl concentration, the adsorption efficiency decreased slightly at 5% (w/v) and increased gradually afterwards. This phenomenon was induced by salting-in effect at low concentration and salting-out effect at higher concentration, which was similar to our previous study [24].

The adsorption capacity of DnPP by imprinted materials (Fe@SiO_2_@MIP) and non-imprinted materials (Fe@SiO_2_@NIP) both decreased from 25°C to 45°C (figure 4*c,d*). They demonstrated no thermal-responsive phenomenon around any temperature, which was in accordance with the study of MIPs by UV–vis spectrophotometer. Compared with the non-imprinted materials, the adsorption capacity of the imprinted materials was about 1.6 times higher when the initial concentration was 2 mg l^−1^ (figure 4*c*), suggesting that molecule imprinted cavity increased the available adsorption sites for DnPP. In addition, the subsequent experimental data showed that the adsorption capacity of Fe@SiO_2_@MIP did not reach the maximum capacity at this time. The adsorption experiment of Fe@SiO_2_@NIP could not be conducted at higher concentration because that data may be unreliable when the final concentration reached the solubility. As a result, it was impractical to make further comparison between Fe@SiO_2_@MIP and Fe@SiO_2_@NIP at higher concentration. It was demonstrated that the adsorption tendency of DnPP by Fe@SiO_2_@MIP at 10 mg l^−1^ (figure 4*d*) was in accordance with that at 2 mg l^−1^ (figure 4*c*). The adsorption capacity was as high as 97.79 mg g^−1^ at 25°C, and decreased about 5% at 45°C.

### 3.3. The adsorption selectivity and competitivity

**Figure 5.**
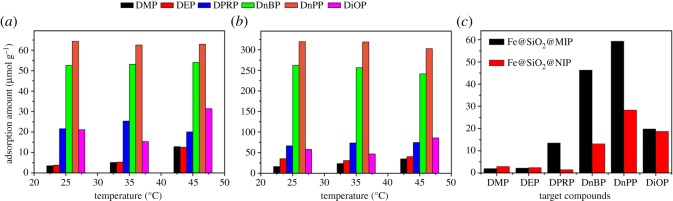
The selective adsorption of six PAEs by Fe@SiO_2_@MIP at different temperatures ((*a*) initial concentration 2 mg l^−1^, (*b*) initial concentration 10 mg l^−1^) and the competitive adsorption of six PAEs by Fe@SiO_2_@MIP and Fe@SiO_2_@NIP at 25°C (*c*).

The selective experiments were carried out in 100 ml vials, containing 2 or 10 mg l^−1^ phthalates (DMP, DEP, DPRP, DnBP, DnPP or DiOP) aqueous solution and 10 mg Fe@SiO_2_@MIP adsorbent, at 25, 35 or 45°C. The vials were agitated for 10 h at 200 r.p.m. and magnetically separated before detection. From figure 5*a*, the adsorption temperature has some influence on the adsorption efficiency. From 25°C to 45°C, adsorption capacity of DnPP and DnBP decreased, that of DiOP showed an increase after a slight decline, while adsorption amount of the other three compounds with shorter alkyl chains increased. In comparison, Fe@SiO_2_@MIP exhibited highest adsorption capacity to target DnPP, and the second highest capacity to DnBP, which had the most similar structure to DnPP (one less methane). Then the adsorption capacities of other substances were much smaller, and the order was as DiOP > DPRP > DEP > DMP. The trend was more obvious when initial concentration was elevated to 10 mg l^−1^ (figure 5*b*). This was a proof that Fe@SiO_2_@MIP had selective adsorption to DnPP, except for the most similar molecule DnBP. The DnPP adsorption amount (319.84 µmol g^−1^) was at least 4.8 times higher than the other phthalates at 25°C when initial concentration was 10 mg l^−1^.

A competitive adsorption conducted when six PAEs coexisted in the same system; 100 ml 2 mg l^−1^ of each PAE (DMP, DEP, DPRP, DnBP, DnPP and DiOP) aqueous solution was added in vials with 10 mg Fe@SiO_2_@MIP or Fe@SiO_2_@NIP adsorbents at 25°C. The supernatants were determined after adsorption and separation processes. From figure 5*c*, adsorption capacity of DnPP by Fe@SiO_2_@MIP was the highest among the six phthalates. DnBP also owned a relatively high amount, 78.06% of DnPP. The adsorption capacity towards different phthalates obeyed the same sequence as the selective study. In a word, DnPP manifested more competitive adsorbability among those phthalates. In contrast to Fe@SiO_2_@MIP, Fe@SiO_2_@NIP obviously exhibited less adsorption capacities towards DnPP, DnBP and DPRP and almost equal adsorbabilities for the other three compounds, which had great difference from DnPP in structure. Capability of Fe@SiO_2_@MIP was 2.1 times higher than Fe@SiO_2_@NIP for the adsorption of DnPP.

### 3.4. Adsorption kinetics

**Figure 6.**
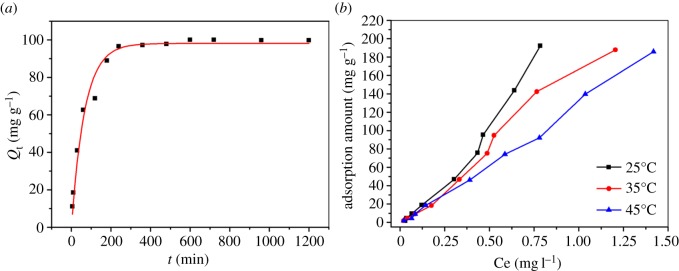
Adsorption kinetics curve of DnPP at 25°C (*a*) and adsorption isotherms of DnPP at 25°C, 35°C and 45°C (*b*) by Fe@SiO_2_@MIP nanocomposites.

The amount of DnPP sorbed onto Fe@SiO_2_@MIP materials with the contact time is presented in figure 6*a*. From this figure, we could see a fast adsorption process occurred during the first 4 h which gradually reached equilibrium within 6 h. The sufficient active sites contributed to the fast adsorption in the initial stage then the adsorption slowed down because there were fewer active sites available. The adsorption capacities under these conditions was determined to be 99.77 mg g^−1^, which was higher than that of similar phthalates by MIP materials observed in He's research [18]. In order to evaluate the kinetics of the adsorption process, the pseudo-first-order, pseudo-second-order and intraparticle diffusion models were tested to interpret the experimental data. The values of constants and correlation coefficients (*R*^2^) are listed in table 1. Data suggested that the pseudo-second-order model was most appropriate, as its correlation coefficient (*R*^2^) was the highest and the calculated adsorption amounts (qe) were in agreement with the actual value. Therefore, the rate-controlling step was the surface adsorption by valence forces through sharing or exchanging of electrons between adsorbent and adsorbate.

**Table 1.** Parameters of adsorption kinetic models.

	parameters		
kinetic models	*k*	*q*_m_	*R*^2^
pseudo-first-order	0.006 (min^−1^)	32.753 (mg g^−1^)	0.545
pseudo-second-order	3.17 × 10^−4^ (g mg^−1^ min^−1^)	103.413 (mg g^−1^)	0.999
intraparticle diffusion	2.609 (mg g^−1^ min^0.5^)	—	0.728

### 3.5. Adsorption isotherm

In order to analyse the adsorption mechanisms, it was imperative to carry out adsorption isotherm studies. The adsorption isotherms of DnPP onto Fe@SiO_2_@MIP at 25°C, 35°C and 45°C are shown in figure 6*b*, indicating an affinity at lower temperature. The maximum equilibrium adsorption amount reached 194.15 mg g^−1^ under optimal conditions. The comparison of adsorption capacities of PAEs with several adsorbents is listed in table 2. The three curves did not reach a plateau till the maximum equilibrium concentration was achieved. Limited by low solubility, the initial concentration could not be set any higher to get a saturation adsorption. Thereafter, the obtained experimental data were fitted to Langmuir, Freundlich, Temkin and D-R isothermal models. The regression parameters are presented in table 3. The processes were better described by the Freundlich model (*R*^2^ = 0.982–0.991) than other models, indicating heterogeneous multilayer adsorption processes. *K*_F_ decreased with the elevation of temperature, which was in agreement with the phenomenon in figure 6*b*. The value of *n* (<0) declared that different adsorption sites existed on the surface of the nanocomposites.

**Table 2.** Maximum adsorption capacity of different adsorbents used for the removal of PAEs.

adsorbent	adsorbent amount (mg)	target PAE	*C*_0_^a^ (mg l^−1^)	adsorption condition	*Q*_m_^b^ (mg g^−1^)	reference
PP-g-CaSiO_3_@SiO_2_	100	DnBP	130	pH 3, 25°C	54.8	[10]
phenyl-functionalized mesoporous silica	20	DnBP	4	pH 7, 25°C	38.9	[11]
molecularly imprinted microspheres	50	DnBP	10	―, 20°C	0.74	[18]
biochar-graphene nanosheet	1	DMP	10	pH 7, 25°C	30.78	[45]
	1	DEP	10	pH 7, 25°C	23.86	
	1	DnBP	4	pH 7, 25°C	21.98	
Fe@SiO_2_@MIP	10	DnBP	10	pH 7, 25°C	73.11	this study
	10	DnPP	20	pH 7, 25°C	194.15	

^a^The initial concentration.

^b^The maximum adsorption amount.

**Table 3.** Parameters of adsorption isotherm models.

isotherm models	temperature (°C)	parameters		
		*K*_L_ (l mg^−1^)	*q*_m_ (mg g^−1^)	*R*^2^
Langmuir	25	0.911	108.696	0.415
	35	0.409	263.158	0.583
	45	0.278	333.333	0.354
		*K*_F_ (l g^−1^)	*n*	*R*^2^
Freundlich	25	251.313	0.775	0.982
	35	170.426	0.863	0.994
	45	131.131	0.889	0.991
		A (l g^−1^)	*b* (J mol^−1^)	*R*^2^
Temkin	25	21.518	52.488	0.776
	35	22.173	61.725	0.764
	45	19.074	65.65	0.788
		$q_{m}^{\prime }$ (mg g^−1^)	*E*_DR_ (kJ mol^−1^)	*R*^2^
D-R	25	148.369	3.162	0.961
	35	110.819	3.162	0.913
	45	104.763	3.162	0.906

### 3.6. Thermodynamic study

The calculated thermodynamic parameters are listed in table 4. The negative values of Δ*G*^0^ indicated the spontaneous property of adsorption. The absolute value of Δ*G*^0^ (less than 20 kJ mol^−1^) in different temperatures suggested physical adsorption processes [33]. Negative Δ*H*^0^ indicated the exothermic nature of adsorption process, which was in accord with the decline tendency of spontaneity from Δ*G*^0^ when temperature was elevated. This was also supported by the decrease of *q*_e_ with upward temperature in solution. The negative value of Δ*S*^0^ revealed the decreasing randomness at the solid–solution interface during the adsorption, which could be explained as the immobilization of DnPP onto adsorbents.

**Table 4.** Parameters of thermodynamics.

	values		
parameters	25°C	35°C	45°C
Δ*G*^0^ (kJ mol^−1^)	−12.233	−11.622	−11.298
Δ*H*^0^ (kJ mol^−1^)		−14.923	
Δ*S*^0^ (kJ mol^−1^ K^−1^)		−0.009	

### 3.7. Reusability of adsorbent

**Figure 7.**
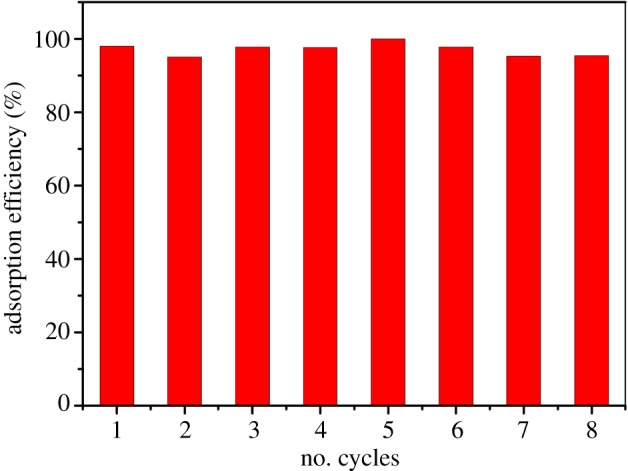
The reusability of the Fe@SiO_2_@MIP adsorbents.

In order to investigate the regeneration ability of the material, the collected 10 mg Fe@SiO_2_@MIP was dispersed in 5 ml methanol and vibrated for 10 min (200 r.p.m.) twice after a 10 h adsorption process in 100 ml 10 mg l^−1^ DnPP aqueous solution at 25°C. The experiment was repeated eight times and the resulting adsorption efficiencies are shown in figure 7. There was no significant decrease in adsorption efficiency in the eight recycles, and the adsorption efficiencies were kept above 95%. It was proved that the material was easy to be regenerated and had strong adsorption ability after regeneration. Besides, the adsorbents exhibited stable chemical properties in experimental conditions.

### 3.8. Analytical method performance

In this experiment, Fe@SiO_2_@MIP materials were also used as MSPE adsorbents to establish an analytical method for DnPP. With the MSPE-HPLC analysis method mentioned above, quantitative parameters were evaluated. The linear range was from 0.5 to 250 µg l^−1^ (*R*^2^ = 0.999). The relative standard deviations (RSD, *n* = 6) was 4.71%. The limits of detection (LOD) was 0.31 µg l^−1^ (*S*/*N *= 3).

**Figure 8.**
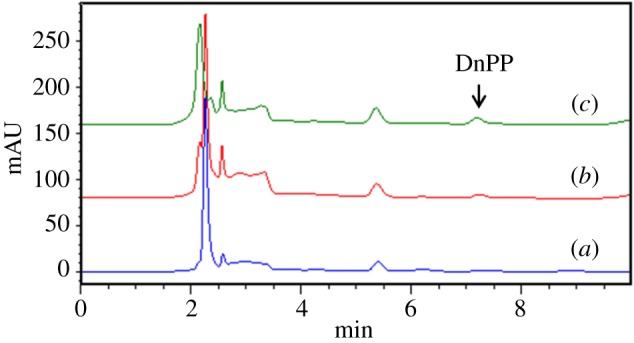
HPLC chromatogram of Changping Park water samples obtained after MSPE with Fe@SiO_2_@MIP at 25°C. (*a*) Blank; (*b*) spiked at 10 µg l^−1^; (*c*) spiked at 20 µg l^−1^.

It was then applied to analyse DnPP in real water samples such as surface water samples and plastic bottles soaking water samples. Ming Tombs Reservoir and Changping Park water samples were collected as the surface water samples. As phthalates are widely used in plastics, the plastic bottles used to containing Wahaha water and Nongfu Spring water were introduced to contain hot water for investigating the release of PAEs. It was found that no target analytes were detected in the water samples. The spiked recoveries of DnPP at two concentration levels (10 and 20 µg l^−1^) were in the range of 94.2–110.4%. The results are shown in table 5. The typical chromatogram of the Changping Park water sample is shown in figure 8. There were significant differences of signal intensity in different spiked samples. From the results above, target DnPP was specifically enriched and remarkably quantified by the established MSPE-HPLC method. The as-prepared Fe@SiO_2_@MIP materials were proved to be potential adsorbents for the adsorption and determination of environmental pollutants.

**Table 5.** Analytical results in real water samples.

	spiked recovery of DnPP (%)^a^			
spiked (μg l^−1^)	Ming Tombs Reservoir	Changping Park water	Wahaha bottle soaked water	Nongfu Spring bottle soaked water
0	n.d.^b^	n.d.	n.d.	n.d.
10	98.9 ± 0.8	110.4 ± 4.2	97.7 ± 0.8	98.3 ± 1.8
20	97.7 ± 3.7	94.2 ± 4.2	104.6 ± 2.6	99.1 ± 3.6

^a^Mean of three determinations ± s. d. (*n* = 3).

^b^Not detected.

## 4. Conclusion

Well-defined magnetic MIP composite materials with stable zero-valent iron as the magnetic core were synthesized in this study. These adsorbents possessed high specific surface area and satisfactory magnetic performance. In the application of Fe@SiO_2_@MIP materials as adsorbents, they exhibited excellent adsorptive capacity towards DnPP as 194.15 mg g^−1^ under experimental condition. Neutral condition and lower temperature (25°C) facilitated the adsorptive process. Meanwhile, Fe@SiO_2_@MIP exhibited certain selectivity adsorption for DnPP. The adsorption amount of DnPP was above 4.8 times higher than that of other tested phthalates, except for DnBP, the one most similar to DnPP in structure. The results demonstrated that the adsorption fitted well with the pseudo-second-order kinetics model and Freundlich isotherm model, which indicated that the surface of the adsorbent was heterogeneous and the active sites were not only the molecular imprinted cavities. The adsorption of DnPP by Fe@SiO_2_@MIP was mainly induced with Van der Waals force, hydrogen bond, hydrophobicity and so on. It was an exothermic spontaneous physical adsorption process. The established MSPE-HPLC method based on Fe@SiO_2_@MIP provided high sensitivity with low LOD for DnPP in water samples. In a word, magnetic nanomaterials Fe@SiO_2_@MIP exhibited their good merits for both removal and determination of DnPP in water, and would probably have a great application prospect in the environmental detection and restoration of other pollutants.

## Supplementary Material

The details of synthesis and equations in adsorption process

## Supplementary Material

Raw data

## Data accessibility

Detailed synthesis process, the equations in adsorption kinetics, isotherms and thermodynamic study are available in the electronic supplementary material.
