# Supplementary material for: Iron-based magnetic molecular imprinted polymers and their application in removal and determination of di-n-pentyl phthalate in aqueous media
Source: R Soc Open Sci. 2017 Aug 16;4(8):170672. doi: 10.1098/rsos.170672 (PMC5579125; doi:10.1098/rsos.170672)
Supplement: The details of synthesis and equations in adsorption process [file rsos170672supp1.docx]

**Supplementary material**

# Iron-based magnetic molecular imprinted polymers (MIPs) and their application in removal and determination of di-n-pentyl phthalate in aqueous media

**Jing Li, Qingxiang Zhou*, Yongyong Yuan, Yalin Wu**

*Beijing Key Laboratory of Oil and Gas Pollution Control,* *College of Geosciences, China University of Petroleum Beijing, Beijing 102249, China*

E-mail: [zhouqx@cup.edu.cn](mailto:zhouqx@cup.edu.cn)

**Table of contents:**

**Part 1. The fabrication of magnetic MIPs composite material 1-3**

**Part 2. The equations in adsorption study 3-6**

**Figure S1.** The UV-Vis transmittance of MIPs solution at different temperatures. **7**

**Part 1. The fabrication of magnetic MIPs composite materials**

**1. Synthesis of Monodisperse Magnetite (Fe_3_O_4_) Particles.**

Firstly, iron-oleate complex was prepared. 10.8 g of iron chloride (FeCl_3_·6H_2_O) and 36.5 g of sodium oleate were dissolved by blowing with a sucker in a mixture solution composed of 60 mL distilled water, 80 mL ethanol and 160 mL hexane. And then the mixture was heated to 70°C and refluxed for 4h. The upper orange organic layer was collected and washed with 30 mL distilled water for three times. Hexane in this organic layer was evaporated. After being dried with vacuum oven, the waxy solid iron–oleate complex was obtained. Secondly, monodisperse magnetite (Fe_3_O_4_) particles were produced by the thermal decomposition method. The obtained iron–oleate complex and 4.3 mL oleic acid were dissolved to 176 mL1-octadecene in a three necked flask at 50-60°C. The reaction mixture was heated to 200 °C with a constant heating rate of 3-4°C min^–1^, and then kept at that temperature for 20 min to dehydration under magnetic agitation. Then the temperature elevated to 320°C at the same rate and kept for 40 min. At that time, a severe reaction occurred and the initial transparent solution turned turbid and brownish black. N_2_ was blown during the procedure. The mixture solution was then cooled naturally to ambient temperature, and nanocrystals in it were precipitated and washed with a mass of ethanol. The obtained Fe_3_O_4_ particles were separated by a magnet and then redispersed in hexane.

**2. Synthesis of Fe@SiO_2_**.

The obtained Fe_3_O_4_ particles were coated with SiO_2_ based on the water-in-cyclohexane reverse microemulsion method. 5.25 mL Fe_3_O_4_ magnetic fluid (68.6 mg/mL in cyclohexane), 14.6 mL Igepal CO-520, 1.52 mL ammonia and 245 mL cyclohexane were mixed and dispersed by magnetic stirring. 30 minutes later, a stable transparent brown solution was obtained. After the addition of 1.72 mL TEOS, the system was kept for 20 h at 25°C. Finally, a large amount of ethanol was poured into the solution to stop the reaction. And then, Fe_3_O_4_@SiO_2_ particles were washed with ethanol for several times and vacuum dried at 50°C. After the formation of Fe_3_O_4_@SiO_2_, a reduction reaction was executed to get Fe@SiO_2_.In particular, Fe_3_O_4_@SiO_2_ particles and four-weight excess of CaH_2_ were finely ground and sealed in a vacuum quartz tube, and heated at 400°C for 48 h. CaH_2_ acted as a reductant in this process. Residual CaH_2_ and CaO produced during the reduction were washed out with an NH_4_Cl/methanol solution and Fe@SiO_2_ particles were separated by magnetic decantation from the system and dried in a vacuum oven at 50°C.

**3. Synthesis of Fe@SiO_2_@MIP and Fe@SiO_2_@NIP**.

Before the thermosensitive modification, MPS was introduced to the surface of Fe@SiO_2_, providing sufficient vinyl groups. 300 mg Fe@SiO_2_, 5 mL MPS and 200 mL ethanol were mixed together and sonicated for 10 min under N_2_. Then the suspension was mechanically stirred for 12 h at 40°C and afterward 0.5 h at 80°C to enhance the band formation. The obtained Fe@SiO_2_-MPS particles were washed with deionized water and dried.

1 mmoL DnPP, 2 mmoL NIPAM and 2 mmoL MAA were dissolved in 100 mL chloroform through adequate magnetic stirring, followed by a pre-polymerization process at 4°C in dark for 12 h. After the temperature of this mixture restored up to room temperature, 0.5 g Fe@SiO_2_-MPS nanoparticles were added under ultrasonic agitation and nitrogen protection. And then, 20 mmoL cross-linker (EGDMA) and 60 mg initiator (AIBN) were added for polymerization with evenly mechanical agitation over 24 h when the temperature reached 60°C. The obtained materials were separated from solution by a magnet and washed with a mixture of methanol and acetic acid (9:1, v/v) several times to remove the template and nonpolymerized residue. Finally, these nanomaterials were dipped in methanol and washed for twice and dried in a vacuum oven overnight at 50°C. The resultant nanomaterials were marked as Fe@SiO_2_@MIP. Meanwhile, the materials without addition of template molecule were synthesized and named as Fe@SiO_2_@NIP.

**Part 2. The equations in adsorption study**

**1. Adsorption kinetics study**

The three conventional adsorption kinetic models of pseudo-first-order model (Eq. (S1)), pseudo-second-order model (Eq. (S2)) and intraparticle diffusion model are as follows (Eq. (S3)):

$ln\left(q_{e}-q_{t}\right)=lnq_{e}-k_{1}t$ (S1)

$\frac{t}{q_{t}}=\frac{1}{k_{2}q_{e}^{2}}+\frac{1}{q_{e}}t$ (S2)

$q_{t}=k_{3}t^{0.5}+C$ (S3)

where k_1_(min^-1^), k_2_ (g mg^-1^ min^-1^) and k_3_ (mg g^-1^ min^0.5^) are the pseudo-first-order, pseudo-second-order and intraparticle diffusion rate constants, while q_e_ and q_t_ (mg g^-1^) are the adsorption capacity at equilibrium and after time t (min), respectively. The value of k_1_, k_2_ and k_3_ were obtained from the slope of the plot of ln(q_e_-q_t_) against t, t/q_t_ against t, and q_t_ against t^0.5^.

**2. Adsorption isotherms and thermodynamic study**

Adsorption isotherm models are detailed below:

Langmuir model:

$\frac{C_{e}}{q_{e}}=\frac{C_{e}}{q_{m}}+\frac{1}{K_{L}q_{m}}$ (S4)

Freundlich model:

$lnq_{e}=\frac{1}{n}lnC_{e}+lnK_{F}$ (S5)

Temkin model:

$q_{e}=BlnA+BlnC_{e}$ (S6)

$B=RT/b$ (S7)

D–R model:

$lnq_{e}=lnq_{m}^{'}-K_{DR}\varepsilon^{2}$ (S8)

$\varepsilon=RTln\left(1+\frac{1}{C_{e}}\right)$ (S9)

$E_{DR}=\left(2K_{DR}\right)^{-0.5}$ (S10)

Where C_e_ is the concentration at equilibrium (mg L^-1^), q_e_ and q_m_ are the adsorption amount at equilibrium and the maximum adsorption capacity (mg g^-1^), respectively. K_L_ (L mg^-1^) is the Langmuir constant. K_F_ (L g^-1^) and n are the Freundlich characteristic constants, reflecting the adsorption capacity and the adsorption intensity, respectively. A (L g^-1^) and b (J mol^-1^) are the equilibrium binding constants corresponding to the maximum binding energy and the Temkin constant, respectively, q_m_’ is the D-R adsorption capacity (mol kg^-1^), K_DR_ is the D-R constant related to the free energy (mol^2^ kJ^-2^) and ε is the Polanyi potential. And E_DR_ (kJ mol^-1^) is the mean free energy of adsorption. In addition, R (8.314 J mol^-1^ K^-1^) is the universal gas constant and T (K) is the absolute solution temperature.

The thermodynamic parameters were calculated from the variation of the thermodynamic equilibrium constant K_0_ with the change in temperature based on following equations:

$K_{0}=\frac{a_{s}}{a_{e}}=\frac{v_{s}}{v_{e}}\times\frac{C_{s}}{C_{e}}$ (S11)

$\Delta G^{0}=-RTlnK_{0}$ (S12)

$lnK_{0}=\frac{\Delta S^{0}}{R}-\frac{\Delta H^{0}}{RT}$ (S13)

$\Delta G^{0}=\Delta H^{0}-T\Delta S^{0}$ (S14)

In these equations, a_s_ is the activity of adsorbed DnPP, a_e_ is the activity of DnPP in solution at equilibrium, ν_s_ is the activity coefficient of the adsorbed DnPP, ν_e_ is the activity coefficient of DnPP in solution, C_s_ is the amount of DnPP adsorbed by per mass of adsorbent (mmol/g), and C_e_ is the concentration of DnPP in solution at equilibrium (mmol/mL). In a dilute solution, lnK_0_ can be calculated from the slope of linear plot ln(Ce/qe) versus qe. Based on the equation of Eq. (S13), ΔH^0^ and ΔS^0^ were obtained from the slope (-ΔH^0^/R) and intercept (ΔS^0^/R) of the ln K_0_ versus 1/T plot.R is the gas constant (8.314 J mol^-1^ K^-1^), and T is the absolute temperature in Kelvin. The Gibbs free energy (ΔG^0^) of adsorption, calculated from the equation of Eq. (S12), is the fundamental criterion to estimate if a reaction is spontaneous at a given temperature from its value. Negative value shows a spontaneous process and the positive one presents an opposite result.





**Figure S1.** The UV-Vis transmittance of MIPs solution at different temperatures.
