# Supplementary material for: Iron-based magnetic molecular imprinted polymers and their application in removal and determination of di-n-pentyl phthalate in aqueous media
Source: R Soc Open Sci. 2017 Aug 16;4(8):170672. doi: 10.1098/rsos.170672 (PMC5579125; doi:10.1098/rsos.170672)
Supplement: Raw data [file rsos170672supp2.docx]

**Raw data**

# Iron-based magnetic molecular imprinted polymers (MIPs) and their application in removal and determination of di-n-pentyl phthalate in aqueous media

**Jing Li, Qingxiang Zhou*, Yongyong Yuan, Yalin Wu**

*Beijing Key Laboratory of Oil and Gas Pollution Control,* *College of Geosciences, China University of Petroleum Beijing, Beijing 102249, China*

E-mail: [zhouqx@cup.edu.cn](mailto:zhouqx@cup.edu.cn)

**Table of contents:**

**1. Influence of pH, ionic concentration and temperature on adsorption efficiency**

**2-3**

**2. The adsorption selectivity and competitivity 4-5**

**3. Adsorption kinetics 5**

**4. Adsorption isotherms 6**

**5. Reusability of adsorbent 7**

**6. Analytical method performance 8**

**1. Influence of pH, ionic concentration and temperature on adsorption efficiency**

| pH influence | | | |
| --- | --- | --- | --- |
| Sample | Peak area 1 | Peak area 2 | Peak area 3 |
| 10 mg L^-1^ DnPP  initial solution | 576.56 |  |  |
| pH 4 | 22.56 | 22.02 | 27.54 |
| pH 5 | 19.44 | 19.36 | 18.38 |
| pH 7 | 12.94 | 12.16 | 13.38 |
| pH 9 | 44.12 | 36.04 | 40.14 |
| pH 11 | 92.36 | 86.18 | 98.48 |

| Ionic concentration influence | | | |
| --- | --- | --- | --- |
| Sample | Peak area 1 | Peak area 2 | Peak area 3 |
| 10 mg L^-1^ DnPP  initial solution | 576.56 |  |  |
| 0% (w/v) NaCl | 14.11 | 15.12 | 12.01 |
| 5% (w/v) NaCl | 22.17 | 24.31 | 19.42 |
| 10% (w/v) NaCl | 8.23 | 9.38 | 6.49 |
| 15% (w/v) NaCl | 6.18 | 7.02 | 5.57 |
| 20% (w/v) NaCl | 0.79 | 1.08 | 0.45 |

| Temperature influence | | | | | |
| --- | --- | --- | --- | --- | --- |
| Sample | | | Peak area 1 | Peak area 2 | Peak area 3 |
| 25°C | 2 mg L^-1^  DnPP | Initial solution | 108.92 |  |  |
|  |  | Fe@SiO_2_@MIP  adsorbed solution | 1.01 | 2.02 | 1.34 |
|  |  | Fe@SiO_2_@NIP  adsorbed solution | 40.34 | 45.12 | 46.50 |
|  | 10 mg L^-1^  DnPP | standard solution | 536.07 |  |  |
|  |  | Fe@SiO_2_@MIP  adsorbed solution | 10.85 | 10.01 | 12.85 |
| 35°C | 2 mg L^-1^ DnPP | initial solution | 112.79 |  |  |
|  |  | Fe@SiO_2_@MIP  adsorbed solution | 4.96 | 3.90 | 4.58 |
|  |  | Fe@SiO_2_@NIP  adsorbed solution | 44.14 | 49.71 | 41.74 |
|  | 10 mg L^-1^  DnPP | standard solution | 544.29 |  |  |
|  |  | Fe@SiO_2_@MIP  adsorbed solution | 10.80 | 14.01 | 11.27 |
| 45°C | 2 mg L^-1^ DnPP | initial solution | 114.81 |  |  |
|  |  | Fe@SiO_2_@MIP  adsorbed solution | 4.36 | 3.34 | 3.95 |
|  |  | Fe@SiO_2_@NIP  adsorbed solution | 46.14 | 46.57 | 56.74 |
|  | 10 mg L^-1^  DnPP | standard solution | 566.24 |  |  |
|  |  | Fe@SiO_2_@MIP  adsorbed solution | 40.98 | 34.84 | 42.34 |

**2. The adsorption selectivity and competitivity**

| Selectivity study | | | | |
| --- | --- | --- | --- | --- |
| Sample | | | Peak area | |
| Condition | | Analyte | Initial solution | Fe@SiO_2_@MIP  adsorbed solution |
| 25°C | 2 mg L^-1^ | DMP | 252.40 | 243.84 |
|  |  | DEP | 226.79 | 217.21 |
|  |  | DPRP | 191.18 | 139.44 |
|  |  | DnBP | 169.57 | 45.41 |
|  |  | DnPP | 108.92 | 1.47 |
|  |  | DiOP | 145.51 | 85.51 |
|  | 10 mg L^-1^ | DMP | 1180.83 | 1144.17 |
|  |  | DEP | 1460.00 | 1345.57 |
|  |  | DPRP | 1030.31 | 858.75 |
|  |  | DnBP | 893.25 | 240.18 |
|  |  | DnPP | 536.07 | 10.85 |
|  |  | DiOP | 857.74 | 663.22 |
| 35°C | 2 mg L^-1^ | DMP | 252.40 | 239.84 |
|  |  | DEP | 244.52 | 230.15 |
|  |  | DPRP | 210.30 | 143.68 |
|  |  | DnBP | 178.32 | 46.49 |
|  |  | DnPP | 112.79 | 4.66 |
|  |  | DiOP | 161.32 | 112.97 |
|  | 10 mg L^-1^ | DMP | 1262.00 | 1204.20 |
|  |  | DEP | 1210.80 | 1127.20 |
|  |  | DPRP | 1032.80 | 842.20 |
|  |  | DnBP | 891.63 | 254.74 |
|  |  | DnPP | 544.29 | 10.80 |
|  |  | DiOP | 779.55 | 636.57 |
| 45°C | 2 mg L^-1^ | DMP | 259.53 | 227.08 |
|  |  | DEP | 258.25 | 222.18 |
|  |  | DPRP | 186.84 | 139.89 |
|  |  | DnBP | 194.81 | 48.19 |
|  |  | DnPP | 114.81 | 3.95 |
|  |  | DiOP | 157.09 | 60.56 |
|  | 10 mg L^-1^ | DMP | 1214.32 | 1132.72 |
|  |  | DEP | 1230.00 | 1120.29 |
|  |  | DPRP | 1032.81 | 839.21 |
|  |  | DnBP | 897.77 | 294.36 |
|  |  | DnPP | 566.24 | 40.98 |
|  |  | DiOP | 780.27 | 517.85 |

| Competitivity study (25°C, 2 mg L^-1^) | | | |
| --- | --- | --- | --- |
| Analyte | Peak area | | |
|  | Initial solution | Fe@SiO_2_@MIP  adsorbed solution | Fe@SiO_2_@NIP  adsorbed solution |
| DMP | 206.72 | 202.79 | 201.01 |
| DEP | 210.45 | 205.50 | 204.94 |
| DPRP | 167.44 | 139.34 | 164.45 |
| DnBP | 146.38 | 52.14 | 119.79 |
| DnPP | 101.52 | 9.36 | 57.44 |
| DiOP | 107.03 | 65.68 | 67.93 |

**3. Adsorption kinetics**

| Adsorption kinetics | | | |
| --- | --- | --- | --- |
| Standard curve | | Sample | |
| Concentration (mg L^-1^) | Peak area | Time (min) | Peak area |
| 0.1 | 7.00 | 5 | 499.30 |
| 0.5 | 30.00 | 10 | 457.88 |
| 1 | 63.64 | 30 | 332.80 |
| 2 | 112.09 | 60 | 212.50 |
| 5 | 287.30 | 120 | 177.68 |
| 10 | 558.81 | 180 | 65.80 |
|  |  | 240 | 22.83 |
|  |  | 360 | 19.83 |
|  |  | 480 | 16.40 |
|  |  | 600 | 3.78 |
|  |  | 720 | 3.92 |
|  |  | 960 | 4.78 |
|  |  | 1200 | 5.02 |

**4. Adsorption isotherms**

| Adsorption isotherms | | | | |
| --- | --- | --- | --- | --- |
|  | Standard curve | | Sample | |
|  | Concentration  (mg L^-1^) | Peak area | Concentration  (mg L^-1^) | Peak area |
| 25°C | 0.01 | 0.50 | 0.2 | 5.46 |
|  | 0.1 | 7.02 | 0.5 | 5.86 |
|  | 1 | 63.64 | 1 | 7.63 |
|  | 2 | 132.09 | 2 | 10.94 |
|  | 5 | 297.30 | 5 | 21.69 |
|  | 10 | 597.52 | 8 | 29.48 |
|  |  |  | 10 | 31.35 |
|  |  |  | 15 | 41.69 |
|  |  |  | 20 | 50.37 |
| 35°C | 0.05 | 4.11 | 0.2 | 5.41 |
|  | 0.1 | 6.79 | 0.5 | 6.55 |
|  | 1 | 60.11 | 1 | 9.07 |
|  | 2 | 117.26 | 2 | 14.24 |
|  | 5 | 298.87 | 5 | 23.02 |
|  | 10 | 561.51 | 8 | 31.77 |
|  |  |  | 10 | 33.92 |
|  |  |  | 15 | 47.45 |
|  |  |  | 20 | 72.22 |
| 45°C | 0.05 | 3.11 | 0.2 | 6.02 |
|  | 0.1 | 8.90 | 0.5 | 8.51 |
|  | 1 | 62.13 | 1 | 9.63 |
|  | 2 | 117.96 | 2 | 12.96 |
|  | 5 | 299.94 | 5 | 26.93 |
|  | 10 | 566.16 | 8 | 38.06 |
|  |  |  | 10 | 49.05 |
|  |  |  | 15 | 63.58 |
|  |  |  | 20 | 85.21 |

**5. Reusability of adsorbent**

| Reusability (25°C, 10 mg L^-1^) | | |
| --- | --- | --- |
| Cycle | Peak area | |
|  | Initial solution | Fe@SiO_2_@MIP  adsorbed solution |
| 1 | 564.62 | 11.28 |
| 2 | 554.38 | 27.64 |
| 3 | 576.56 | 12.94 |
| 4 | 590.24 | 14.11 |
| 5 | 598.81 | 0.14 |
| 6 | 541.88 | 11.98 |
| 7 | 597.52 | 27.76 |
| 8 | 519.22 | 23.83 |

**6. Analytical method performance**

| Analytical method | | |
| --- | --- | --- |
| Linear range | | RSD (C_0_=10 μg L^-1^) |
| Concentration  (μg L^-1^) | Peak area | Peak area |
| 0.5 | 15.46 | 64.30 |
| 2 | 18.75 | 64.10 |
| 20 | 124.05 | 63.50 |
| 100 | 518.33 | 65.00 |
| 200 | 991.39 | 64.30 |
| 250 | 1187.79 | 71.70 |

| Determination of spiked real water samples | | | | | |
| --- | --- | --- | --- | --- | --- |
| Real sample | Spiked concentration  (μg L^-1^) | Peak area | | | |
|  |  | Distilled water | Sample 1 | Sample 2 | Sample 3 |
| Ming Tombs Reservoir | 10 | 65.56 | 63.92 | 65.20 | 65.50 |
|  | 20 | 124.48 | 122.12 | 117.69 | 125.01 |
| Changping Park water | 10 | 58.76 | 64.01 | 64.97 | 71.75 |
|  | 20 | 127.51 | 117.69 | 125.01 | 117.75 |
| Wahaha bottle soaked water | 10 | 65.75 | 64.10 | 65.09 | 63.50 |
|  | 20 | 107.73 | 112.61 | 110.20 | 115.30 |
| Nongfu Spring bottle soaked water | 10 | 88.54 | 88.60 | 85.00 | 87.50 |
|  | 20 | 135.10 | 134.07 | 130.20 | 137.51 |
